# Supplementary material for: Meru co-ordinates spindle orientation with cell polarity and cell cycle progression
Source: EMBO J. 2025 Apr 1;44(10):2949–75. doi: 10.1038/s44318-025-00420-5 (PMC12084343; doi:10.1038/s44318-025-00420-5)
Supplement: Supplementary file 2 — Movie EV1 [file 44318_2025_420_MOESM2_ESM.zip › EV Movie 1/EV Movie 1 figure legend.rtf]

Title: Wild-type SOPs divide along the A-P axis Description: Confocal live-imaging of an SOP division in the pupal notum at 16 h APF. SOPs are marked by neur-H2B-RFP (magenta) and the spindle is marked by Jupiter-GFP (green). The spindle aligns along the A-P axis and the daughter cells are segregated anteriorly and posteriorly (top and bottom of frame, respectively). Scale bar = 10 μ
