# Supplementary material for: Meru co-ordinates spindle orientation with cell polarity and cell cycle progression
Source: EMBO J. 2025 Apr 1;44(10):2949–75. doi: 10.1038/s44318-025-00420-5 (PMC12084343; doi:10.1038/s44318-025-00420-5)
Supplement: Supplementary file 3 — Movie EV2 [file 44318_2025_420_MOESM3_ESM.zip › EV Movie 2/EV Movie 2 figure legend.rtf]

Title: Loss of meru leads to spindle misalignment during SOP division Description: Confocal live-imaging of an SOP division in the meru1 mutant background in the pupal notum at 16 h APF. SOPs are marked by neur-H2B-RFP (magenta) and the spindle is marked by Jupiter-GFP (green). In this example, the spindle aligns perpendicular to the A-P axis (top to bottom of frame). Scale bar = 10 μ.
