# Supplementary material for: Meru co-ordinates spindle orientation with cell polarity and cell cycle progression
Source: EMBO J. 2025 Apr 1;44(10):2949–75. doi: 10.1038/s44318-025-00420-5 (PMC12084343; doi:10.1038/s44318-025-00420-5)
Supplement: Supplementary file 4 — Source data Fig. 1 [file 44318_2025_420_MOESM4_ESM.zip › Fig1/1F/READ ME.rtf]

ImageJ Processing:RemoveOutliers: 2.00GaussianBlur: 0.5
