# Supplementary figures and images for: Meru co-ordinates spindle orientation with cell polarity and cell cycle progression
Source: EMBO J. 2025 Apr 1;44(10):2949–75. doi: 10.1038/s44318-025-00420-5 (PMC12084343; doi:10.1038/s44318-025-00420-5)

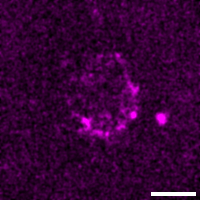

Supplement: Supplementary file 4 — Source data Fig. 1 [file 44318_2025_420_MOESM4_ESM.zip › Fig1/1F/ImageData Meru.tif]

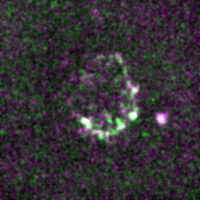

Supplement: Supplementary file 4 — Source data Fig. 1 [file 44318_2025_420_MOESM4_ESM.zip › Fig1/1F/ImageData Merged.tif]

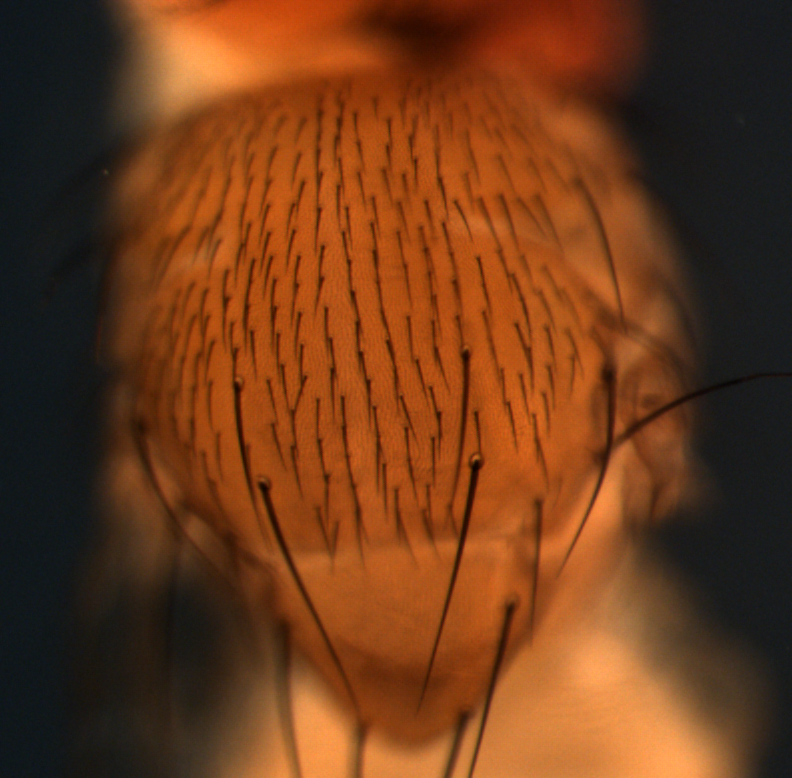

Supplement: Supplementary file 4 — Source data Fig. 1 [file 44318_2025_420_MOESM4_ESM.zip › Fig1/1B/ImageData.tif]

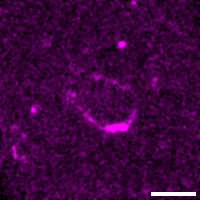

Supplement: Supplementary file 4 — Source data Fig. 1 [file 44318_2025_420_MOESM4_ESM.zip › Fig1/1E/ImageData Meru.tif]

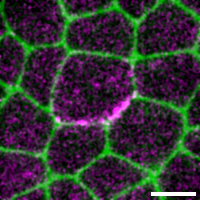

Supplement: Supplementary file 4 — Source data Fig. 1 [file 44318_2025_420_MOESM4_ESM.zip › Fig1/1D/ImageData.tif]

**Fig 2C**

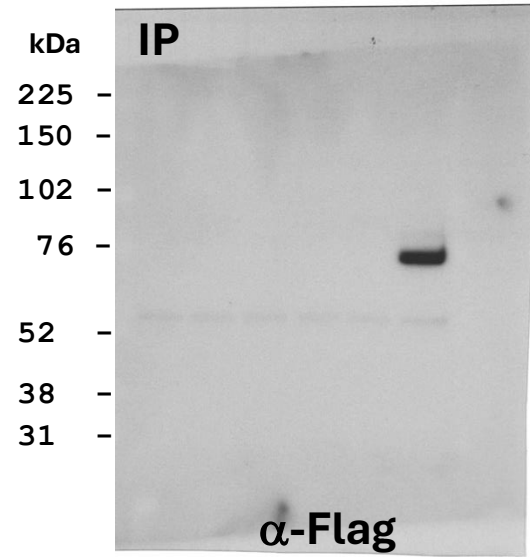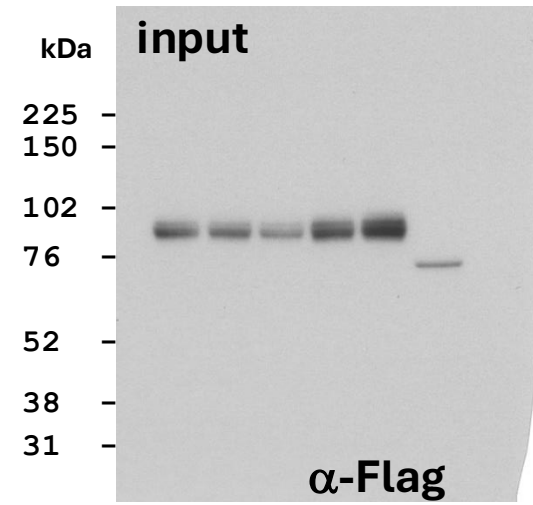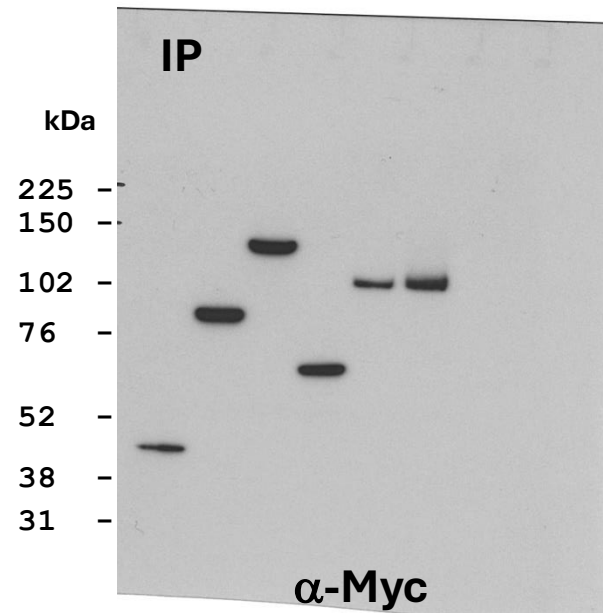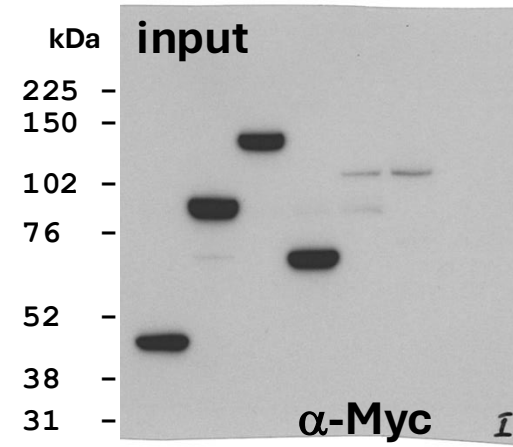

Supplement: Supplementary file 5 — Source data Fig. 2 [file 44318_2025_420_MOESM5_ESM.zip › Fig2/2C/Fig 2C.pdf]

**Fig 2D**

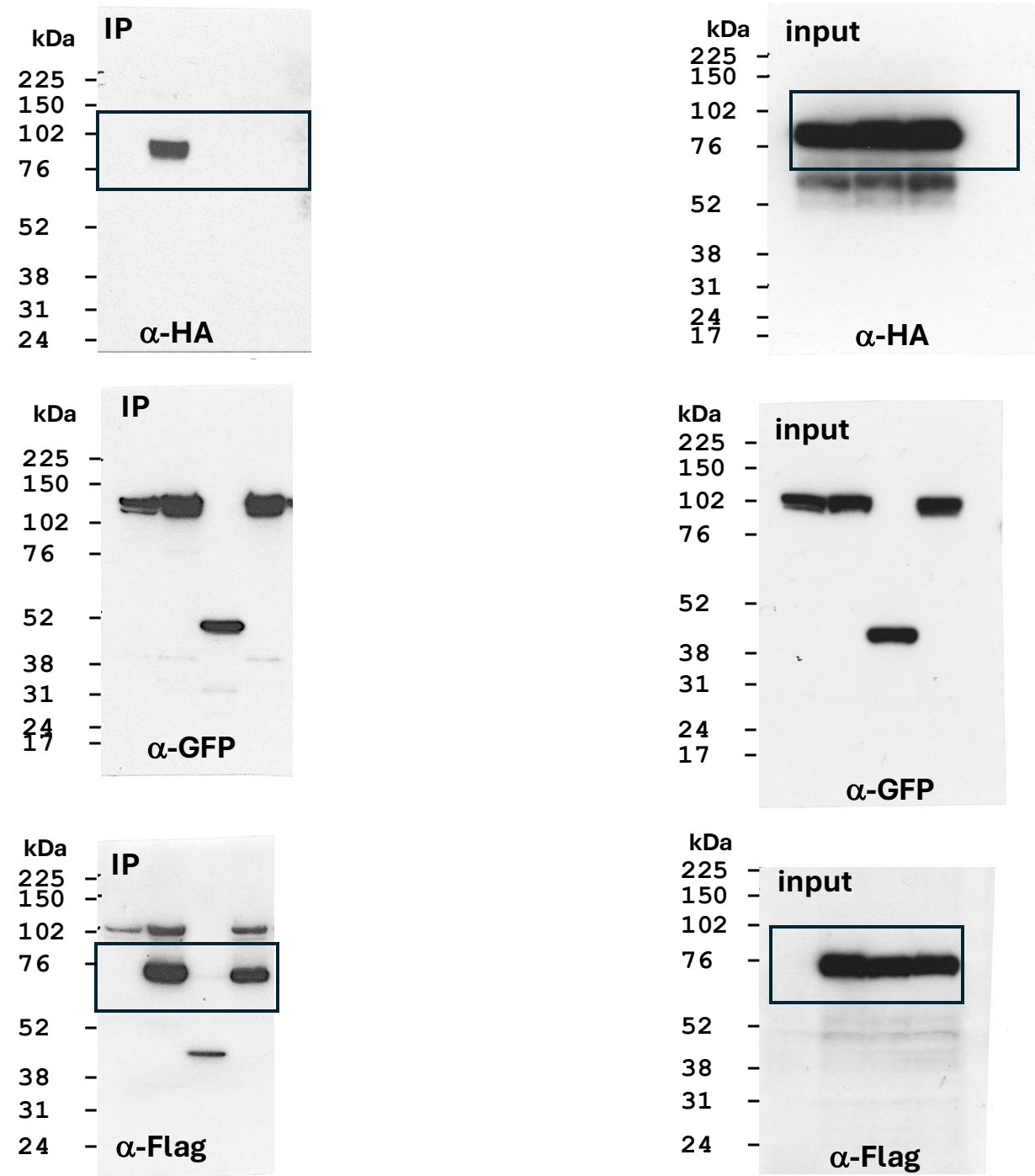

Supplement: Supplementary file 5 — Source data Fig. 2 [file 44318_2025_420_MOESM5_ESM.zip › Fig2/2D/Fig 2D.pdf]

**Fig 2B**

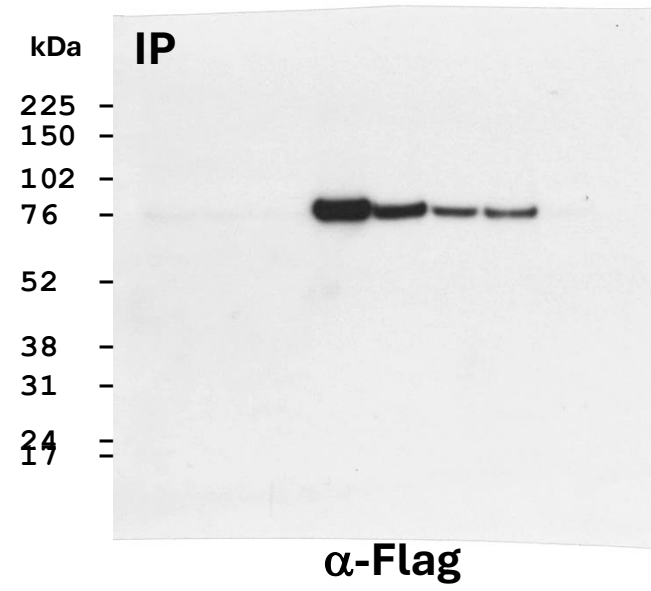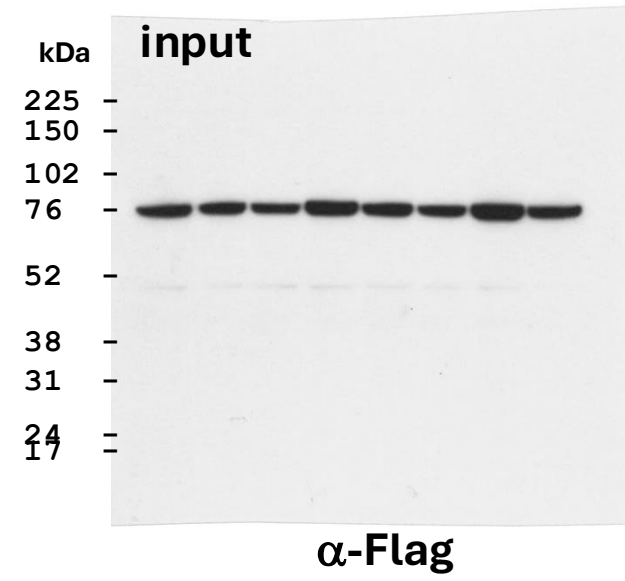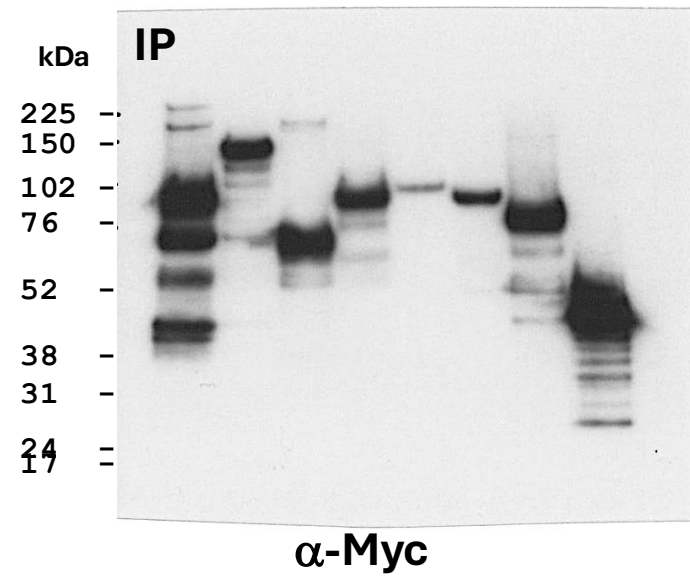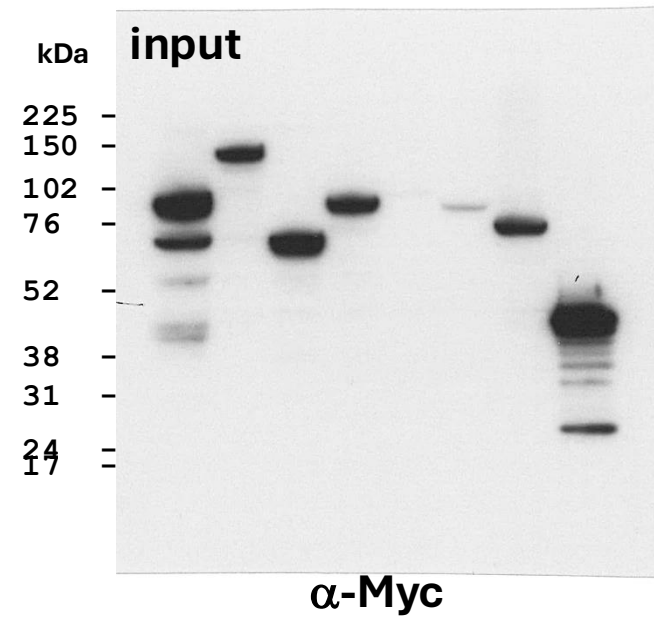

Supplement: Supplementary file 5 — Source data Fig. 2 [file 44318_2025_420_MOESM5_ESM.zip › Fig2/2B/Fig 2B.pdf]

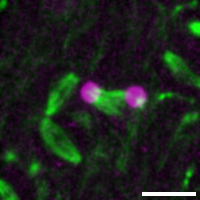

Supplement: Supplementary file 6 — Source data Fig. 3 [file 44318_2025_420_MOESM6_ESM.zip › Fig3/3E/ImageData.tif]

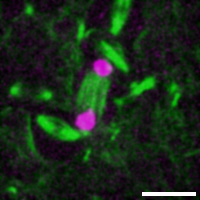

Supplement: Supplementary file 6 — Source data Fig. 3 [file 44318_2025_420_MOESM6_ESM.zip › Fig3/3D/ImageData.tif]

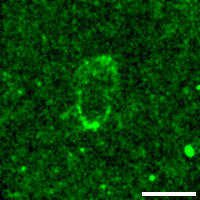

Supplement: Supplementary file 6 — Source data Fig. 3 [file 44318_2025_420_MOESM6_ESM.zip › Fig3/3A/ImageData Mud.tif]

Fig 4C

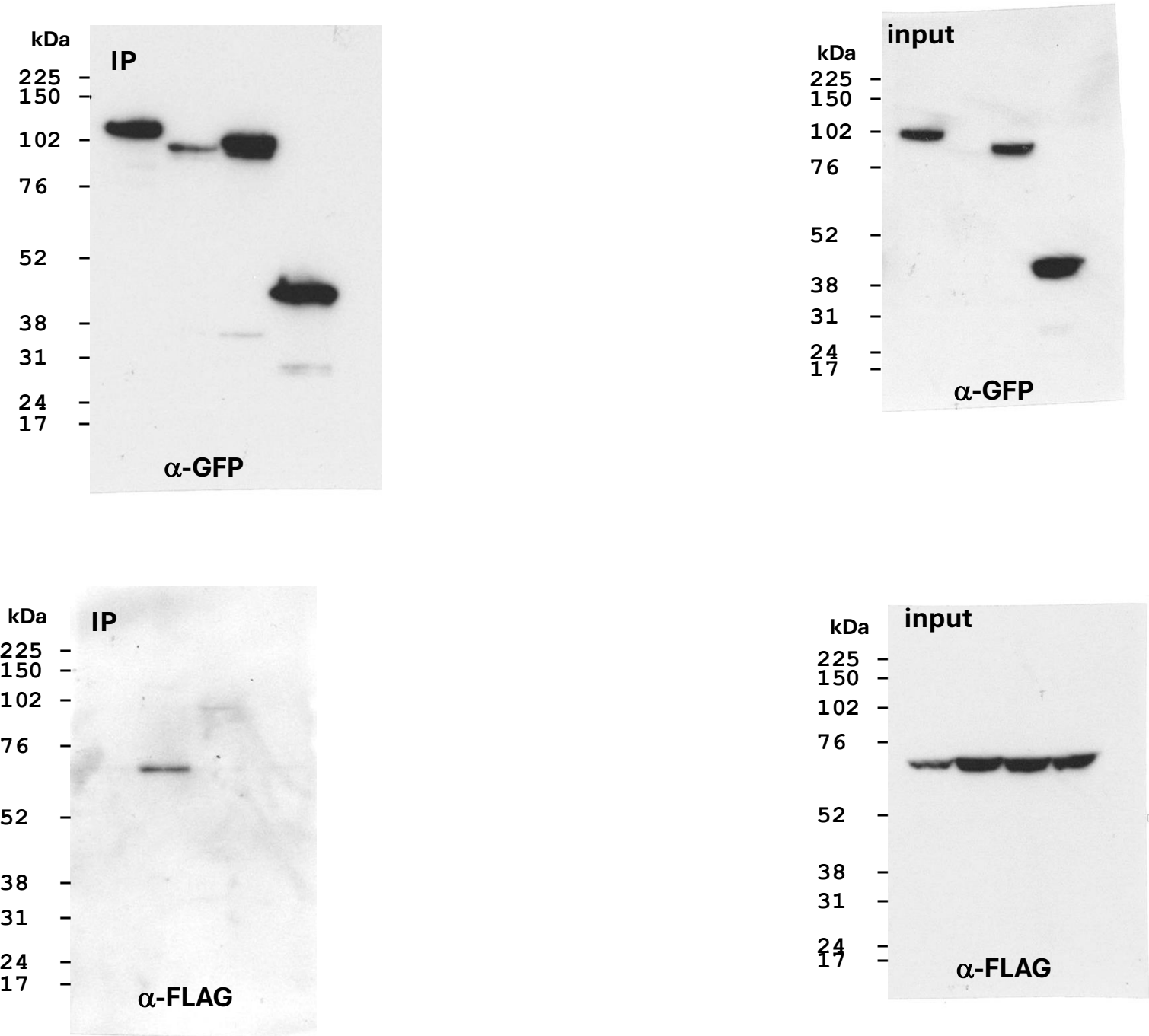

Supplement: Supplementary file 7 — Source data Fig. 4 [file 44318_2025_420_MOESM7_ESM.zip › Fig4/4C/Fig 4C.pdf]

Fig 4D

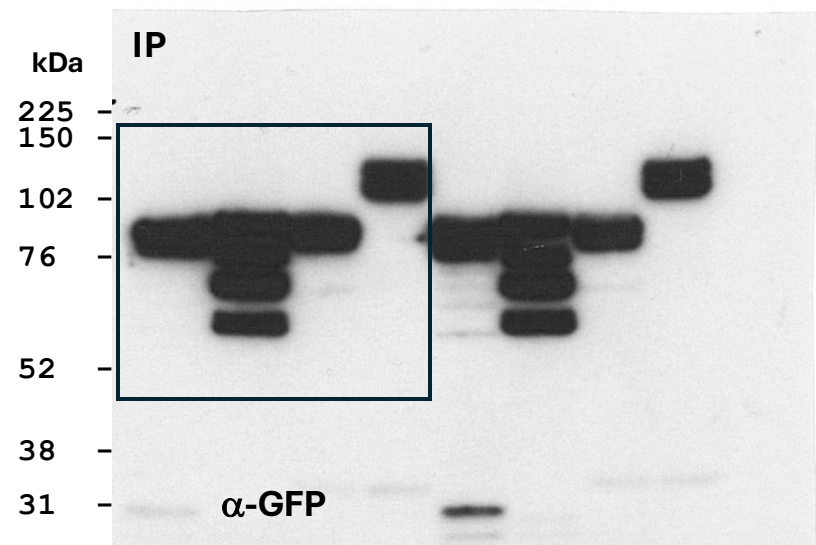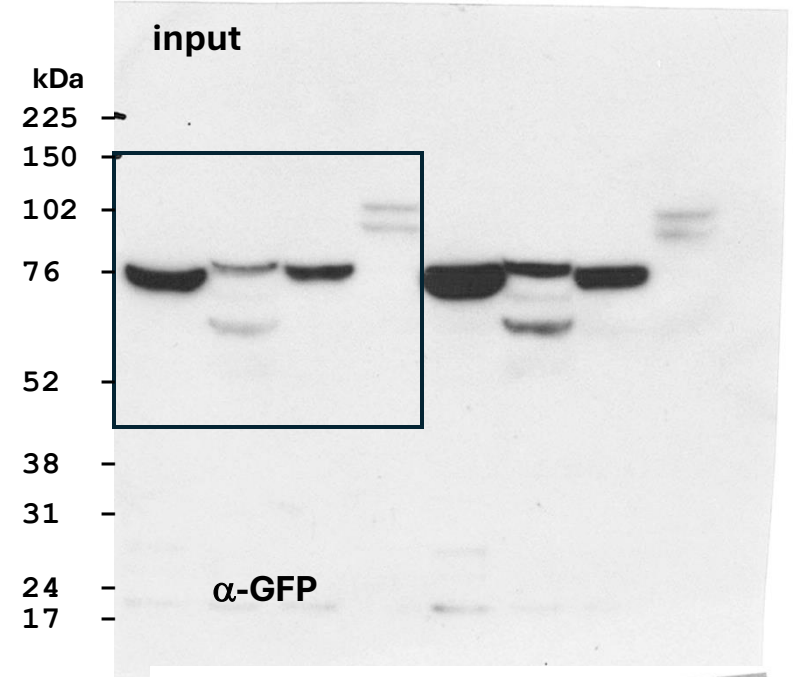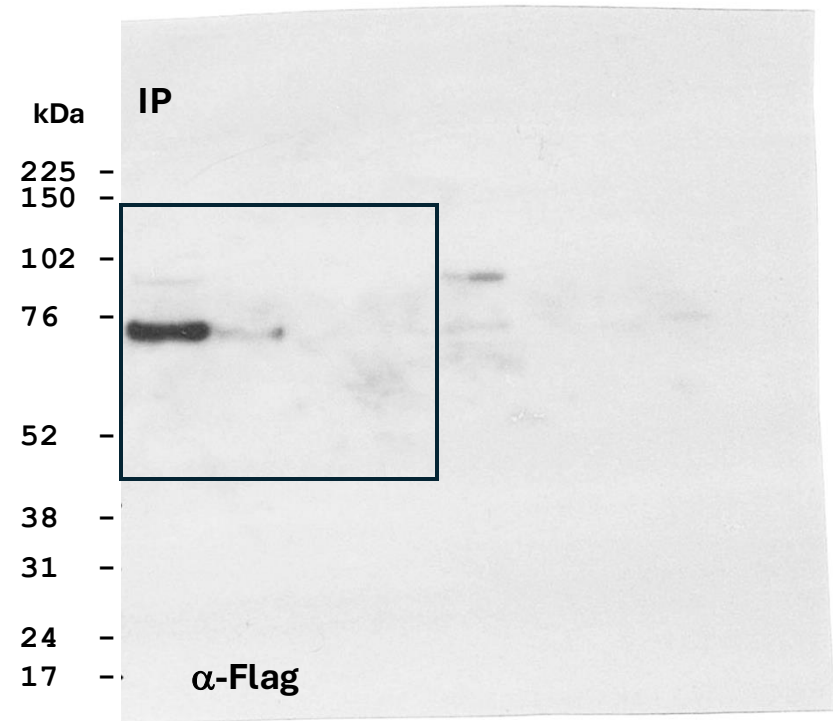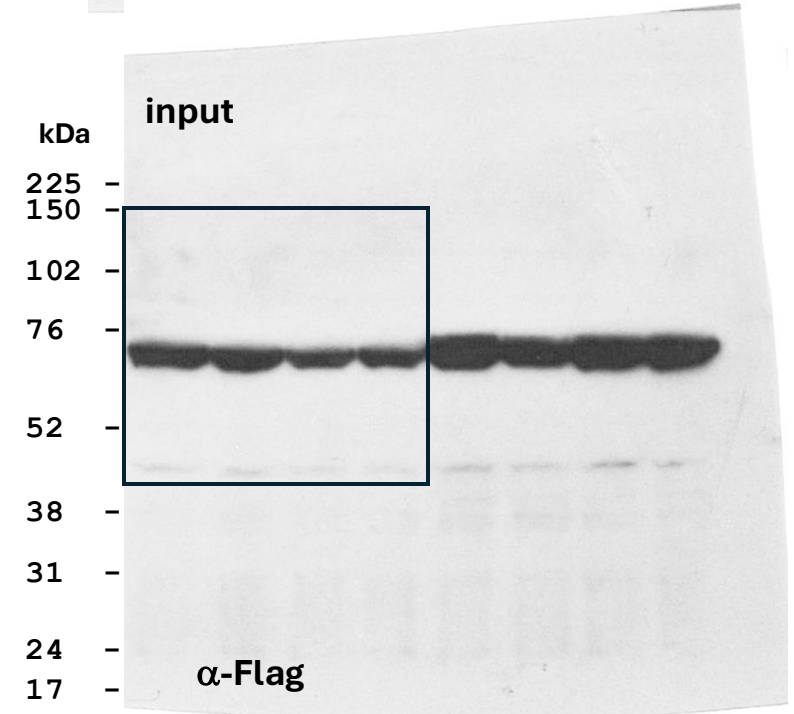

Supplement: Supplementary file 7 — Source data Fig. 4 [file 44318_2025_420_MOESM7_ESM.zip › Fig4/4D/Fig 4D.pdf]

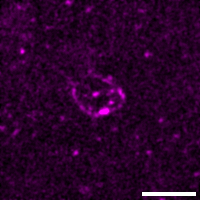

Supplement: Supplementary file 7 — Source data Fig. 4 [file 44318_2025_420_MOESM7_ESM.zip › Fig4/4A/ImageData Meru -20mins.tif]

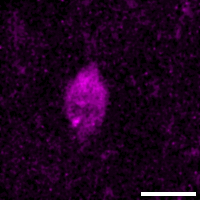

Supplement: Supplementary file 8 — Source data Fig. 5 [file 44318_2025_420_MOESM8_ESM.zip › Fig5/5F/ImageData Meru -18mins.tif]

Fig 5C

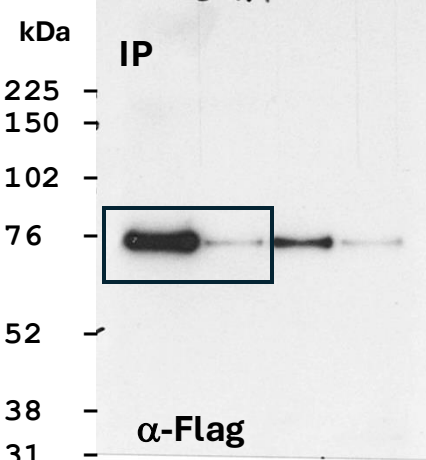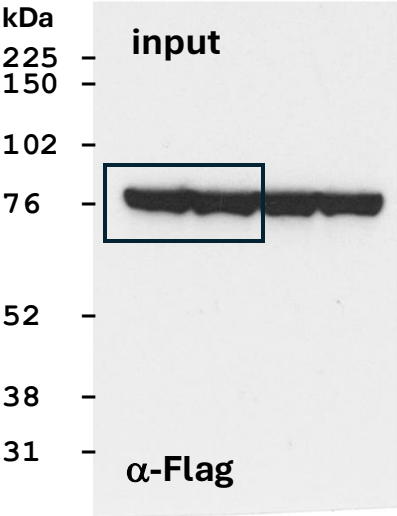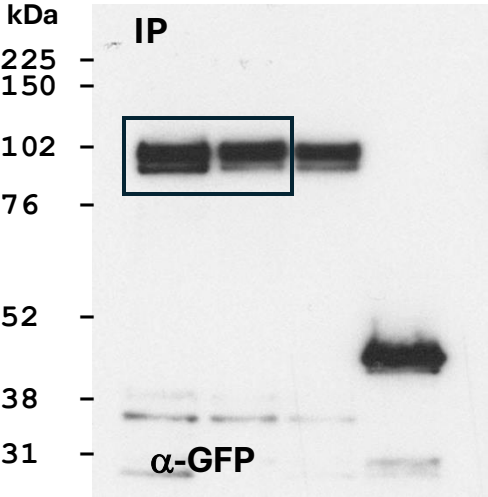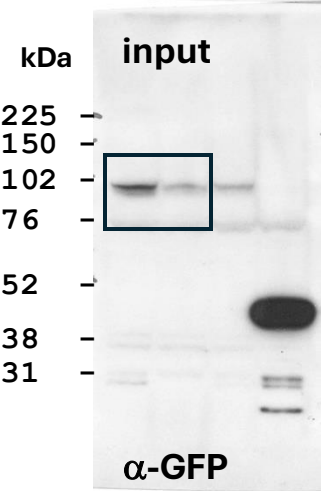

Supplement: Supplementary file 8 — Source data Fig. 5 [file 44318_2025_420_MOESM8_ESM.zip › Fig5/5C/Fig 5C.pdf]

Fig 5D

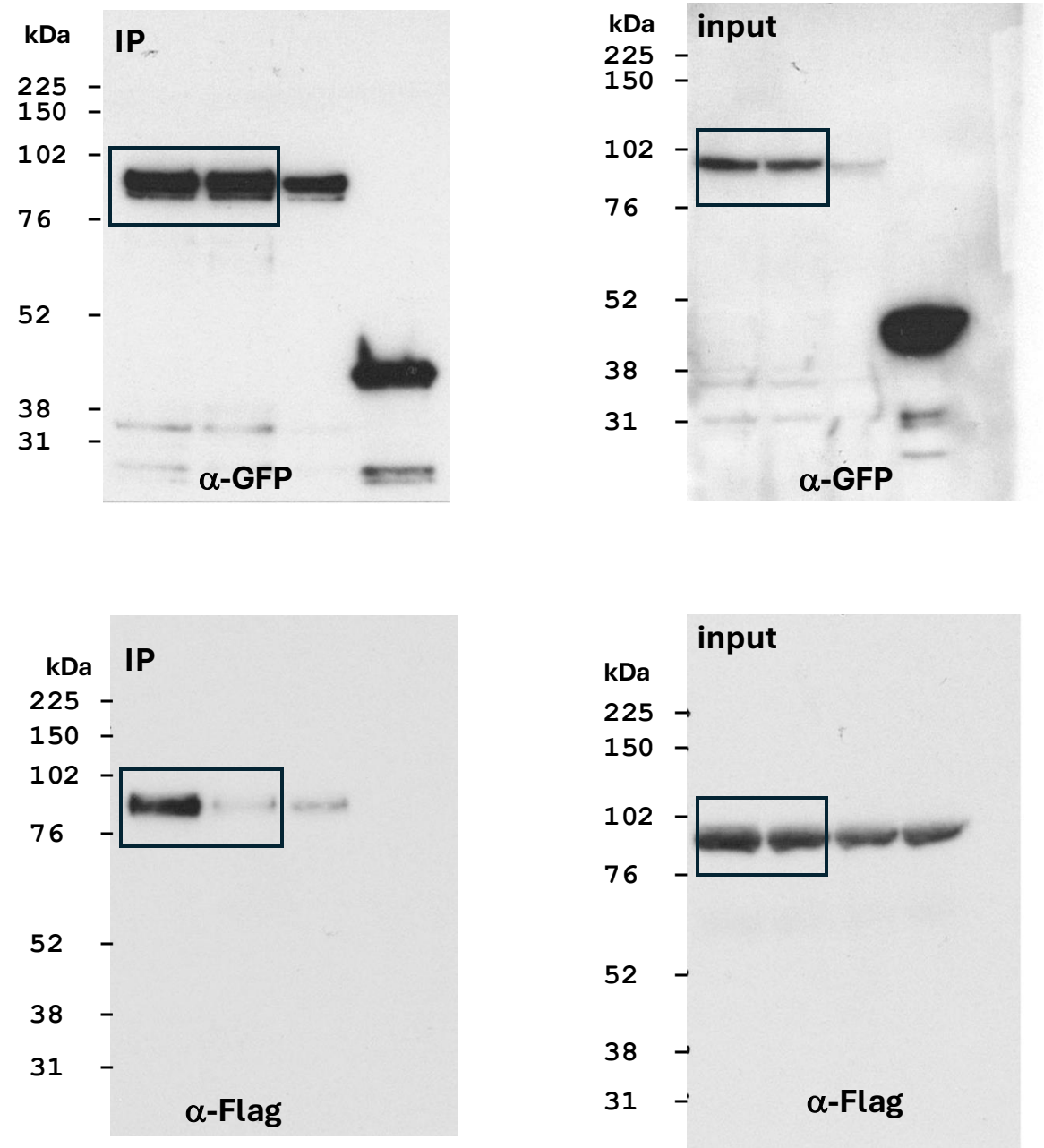

Supplement: Supplementary file 8 — Source data Fig. 5 [file 44318_2025_420_MOESM8_ESM.zip › Fig5/5D/Fig 5D.pdf]

Fig 5E

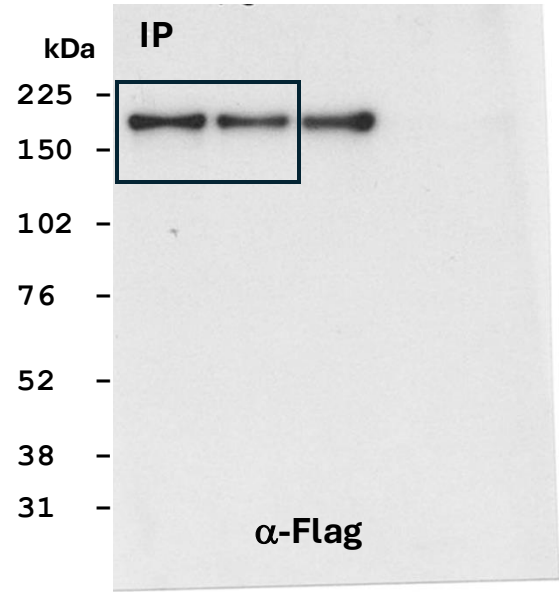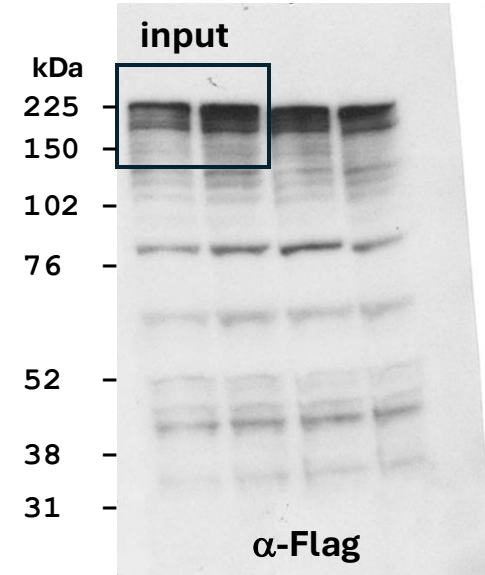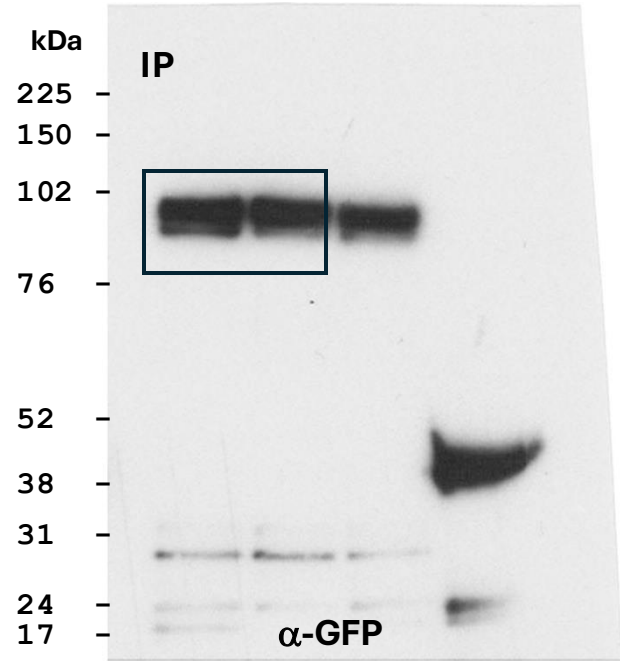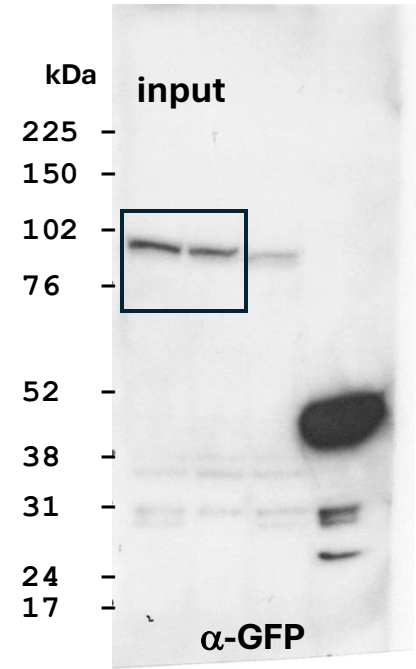

Supplement: Supplementary file 8 — Source data Fig. 5 [file 44318_2025_420_MOESM8_ESM.zip › Fig5/5E/Fig 5E.pdf]

**Fig 5B**

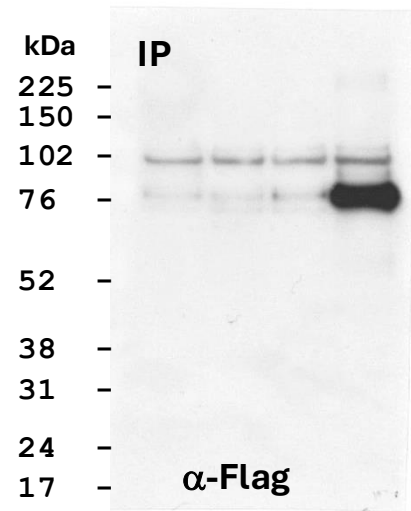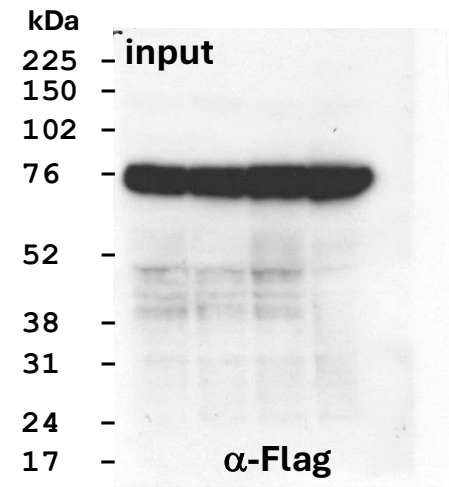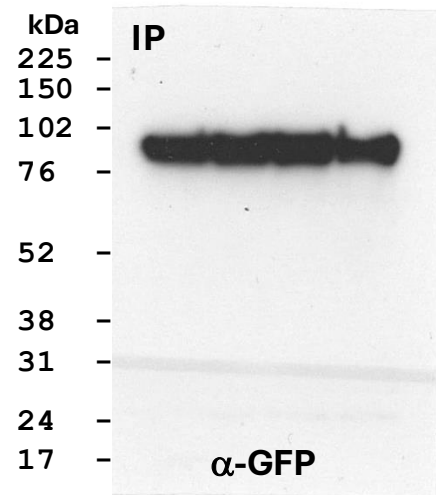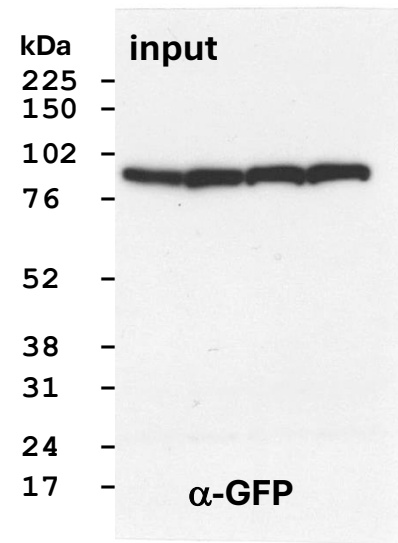

Supplement: Supplementary file 8 — Source data Fig. 5 [file 44318_2025_420_MOESM8_ESM.zip › Fig5/5B/Fig 5B.pdf]

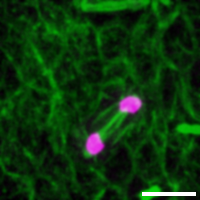

Supplement: Supplementary file 9 — Source data Fig. 6 [file 44318_2025_420_MOESM9_ESM.zip › Fig6/6F/ImageData.tif]

Fig 6

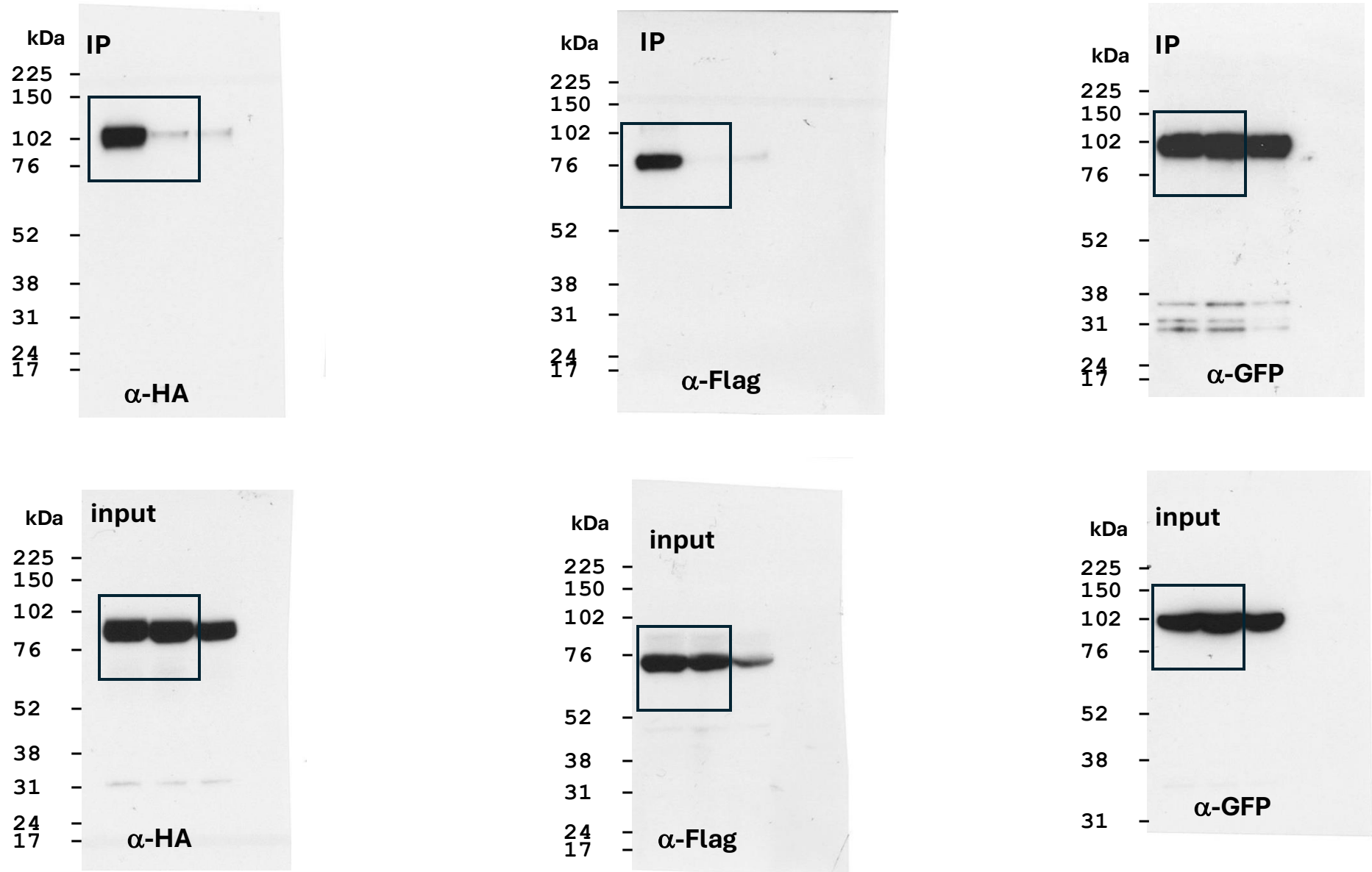

Supplement: Supplementary file 9 — Source data Fig. 6 [file 44318_2025_420_MOESM9_ESM.zip › Fig6/6A/Fig 6A.pdf]

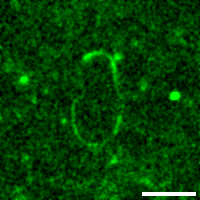

Supplement: Supplementary file 9 — Source data Fig. 6 [file 44318_2025_420_MOESM9_ESM.zip › Fig6/6B/ImageData Mud.tif]

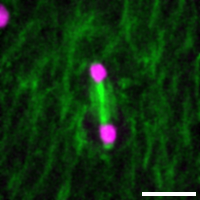

Supplement: Supplementary file 9 — Source data Fig. 6 [file 44318_2025_420_MOESM9_ESM.zip › Fig6/6E/ImageData.tif]

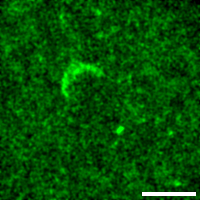

Supplement: Supplementary file 9 — Source data Fig. 6 [file 44318_2025_420_MOESM9_ESM.zip › Fig6/6C/ImageData Mud.tif]

Fig 7D

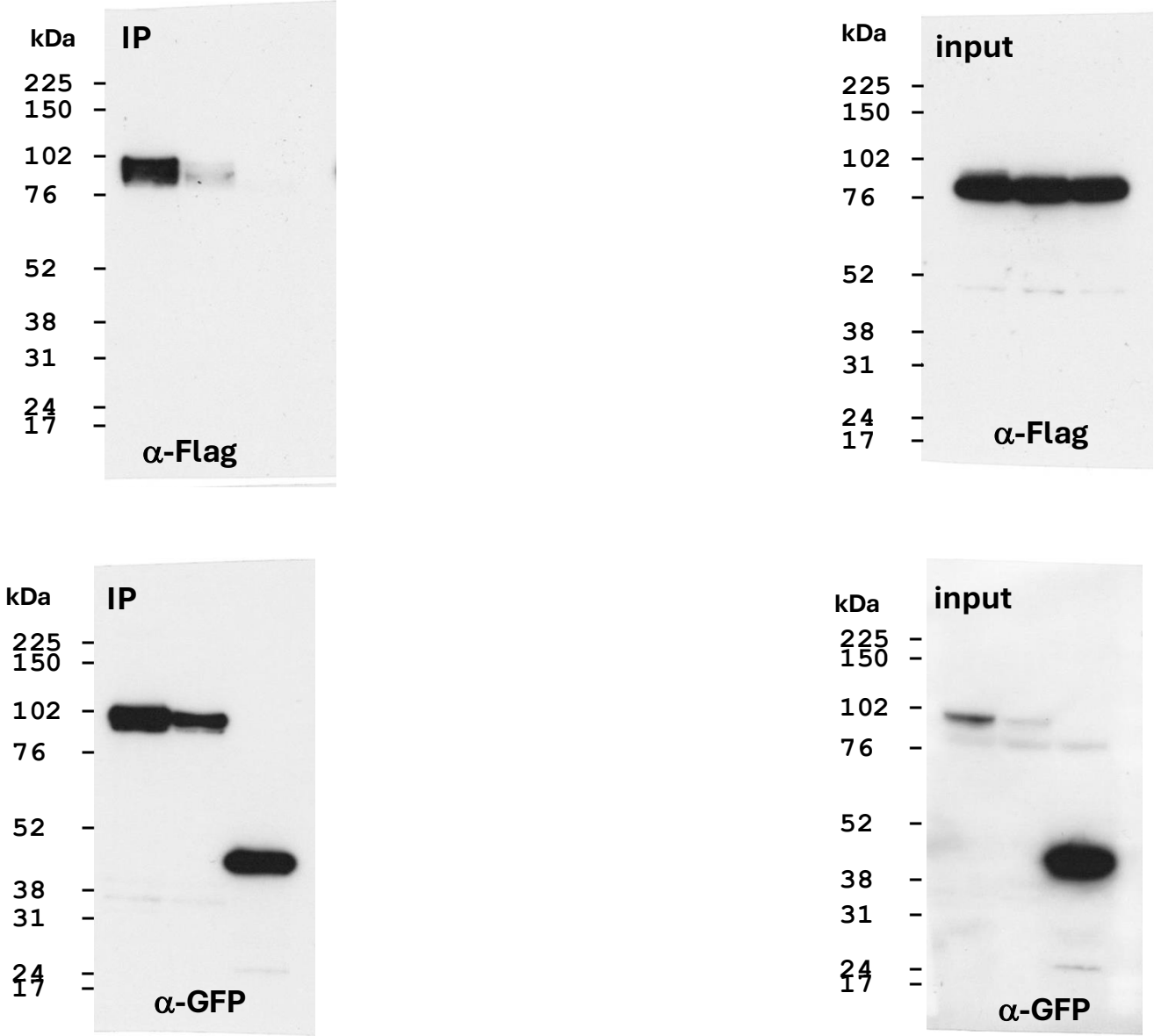

Supplement: Supplementary file 10 — Source data Fig. 7 [file 44318_2025_420_MOESM10_ESM.zip › Fig7/7D/Fig 7D.pdf]

**Fig 7C**

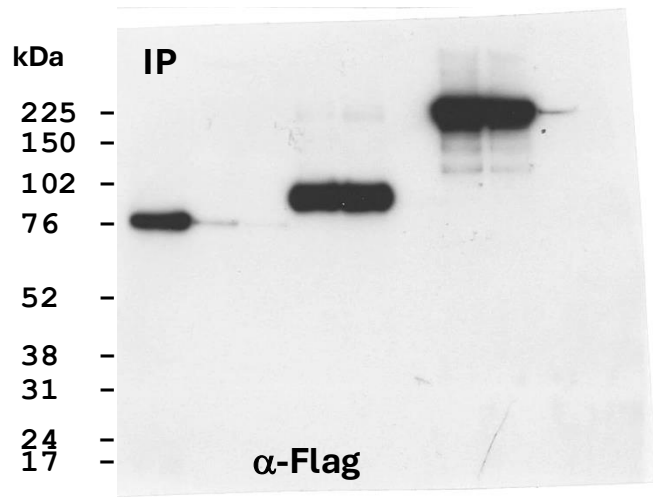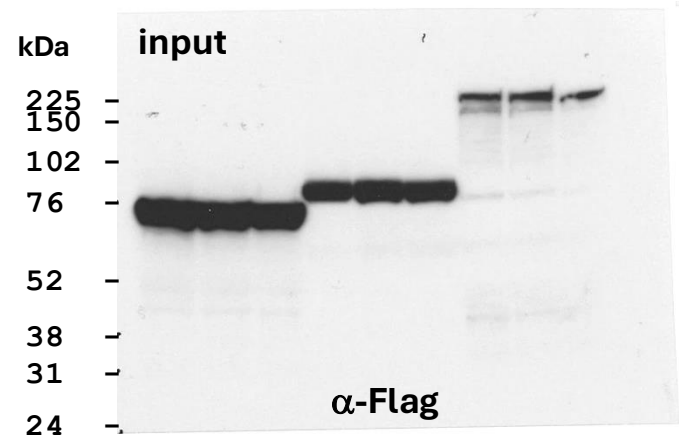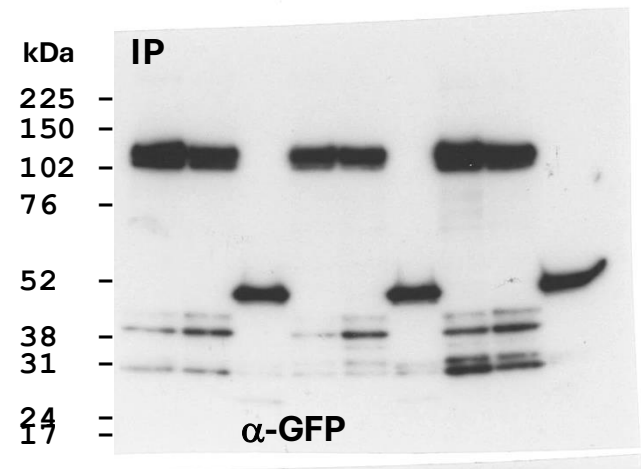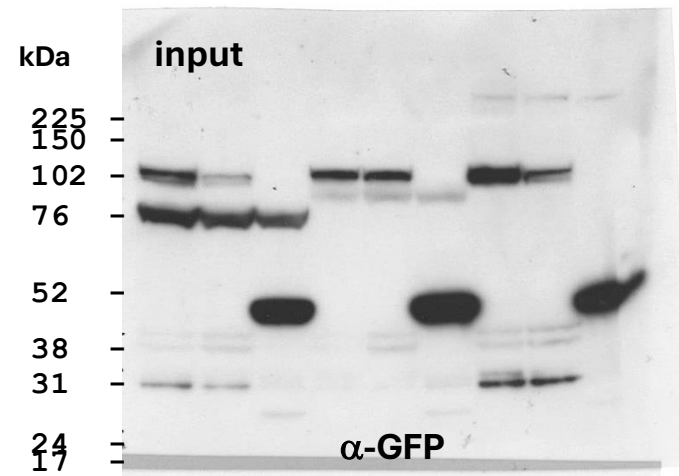

Supplement: Supplementary file 10 — Source data Fig. 7 [file 44318_2025_420_MOESM10_ESM.zip › Fig7/7C/Fig 7C.pdf]

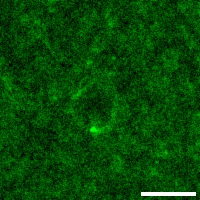

Supplement: Supplementary file 11 — Source data Fig. 8 [file 44318_2025_420_MOESM11_ESM.zip › Fig8/8A/ImageData Mud.tif]

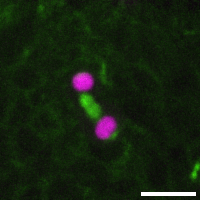

Supplement: Supplementary file 11 — Source data Fig. 8 [file 44318_2025_420_MOESM11_ESM.zip › Fig8/8D/ImageData.tif]

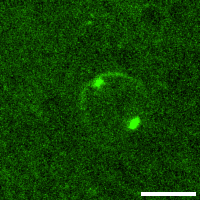

Supplement: Supplementary file 11 — Source data Fig. 8 [file 44318_2025_420_MOESM11_ESM.zip › Fig8/8B/ImageData Mud.tif]

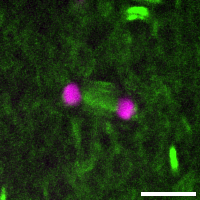

Supplement: Supplementary file 11 — Source data Fig. 8 [file 44318_2025_420_MOESM11_ESM.zip › Fig8/8E/ImageData.tif]

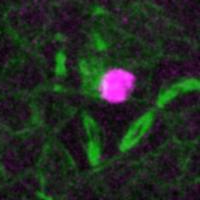

Supplement: Supplementary file 12 — EV Figure Source Data [file 44318_2025_420_MOESM12_ESM.zip › Source_data_EV/EV Movie 1/ImageData.tif]

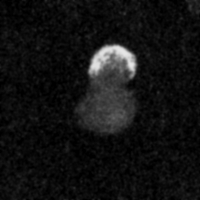

Supplement: Supplementary file 12 — EV Figure Source Data [file 44318_2025_420_MOESM12_ESM.zip › Source_data_EV/EV6/6B/ImageData 0mins.tif]

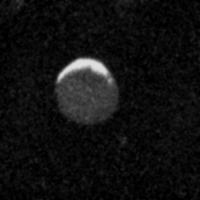

Supplement: Supplementary file 12 — EV Figure Source Data [file 44318_2025_420_MOESM12_ESM.zip › Source_data_EV/EV6/6B/ImageData -6mins.tif]

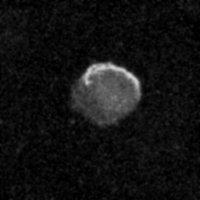

Supplement: Supplementary file 12 — EV Figure Source Data [file 44318_2025_420_MOESM12_ESM.zip › Source_data_EV/EV6/6B/ImageData -18mins.tif]

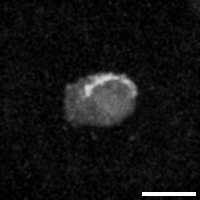

Supplement: Supplementary file 12 — EV Figure Source Data [file 44318_2025_420_MOESM12_ESM.zip › Source_data_EV/EV6/6B/ImageData -24mins.tif]

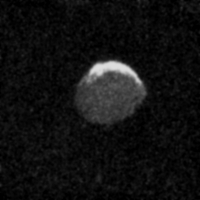

Supplement: Supplementary file 12 — EV Figure Source Data [file 44318_2025_420_MOESM12_ESM.zip › Source_data_EV/EV6/6B/ImageData -12mins.tif]

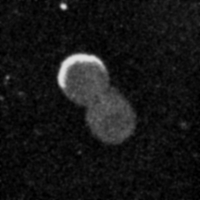

Supplement: Supplementary file 12 — EV Figure Source Data [file 44318_2025_420_MOESM12_ESM.zip › Source_data_EV/EV6/6C/ImageData 0mins.tif]

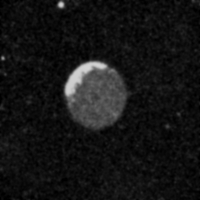

Supplement: Supplementary file 12 — EV Figure Source Data [file 44318_2025_420_MOESM12_ESM.zip › Source_data_EV/EV6/6C/ImageData -6mins.tif]

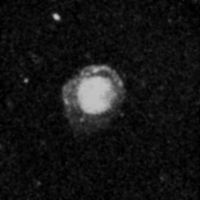

Supplement: Supplementary file 12 — EV Figure Source Data [file 44318_2025_420_MOESM12_ESM.zip › Source_data_EV/EV6/6C/ImageData -18mins.tif]

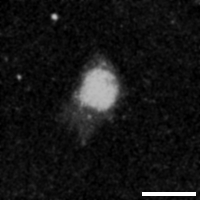

Supplement: Supplementary file 12 — EV Figure Source Data [file 44318_2025_420_MOESM12_ESM.zip › Source_data_EV/EV6/6C/ImageData -24mins.tif]

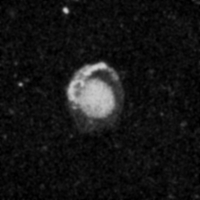

Supplement: Supplementary file 12 — EV Figure Source Data [file 44318_2025_420_MOESM12_ESM.zip › Source_data_EV/EV6/6C/ImageData -12mins.tif]

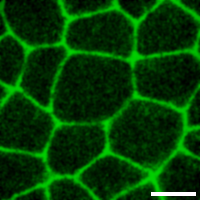

Supplement: Supplementary file 12 — EV Figure Source Data [file 44318_2025_420_MOESM12_ESM.zip › Source_data_EV/EV1/1A/ImageData Ecad -24mins.tif]

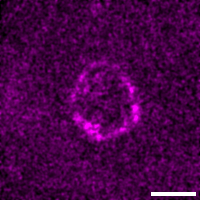

Supplement: Supplementary file 12 — EV Figure Source Data [file 44318_2025_420_MOESM12_ESM.zip › Source_data_EV/EV1/1B/ImageData Meru -12mins.tif]

EV2 C

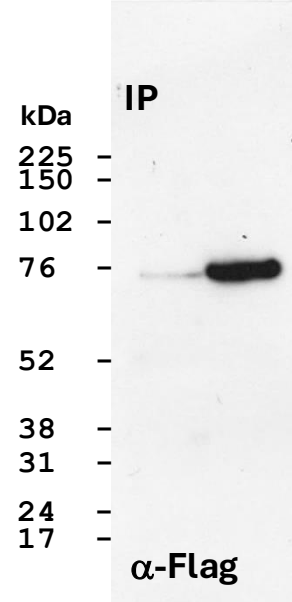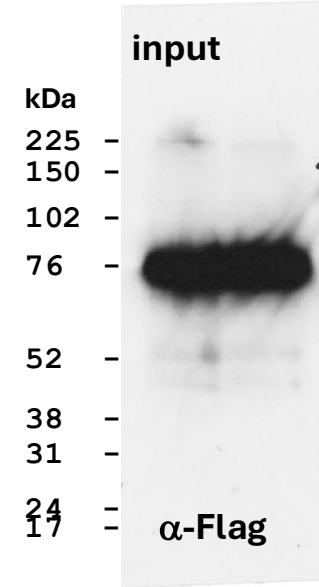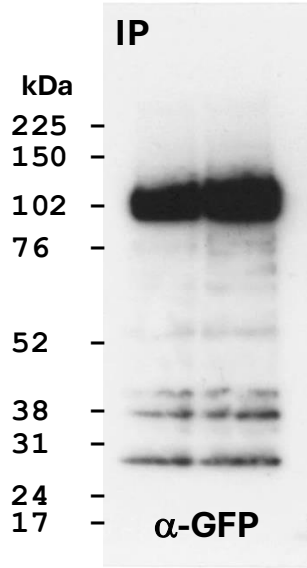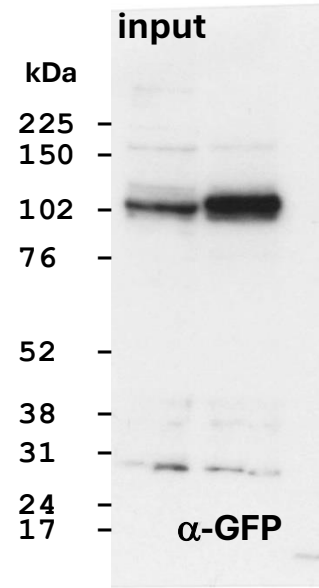

Supplement: Supplementary file 12 — EV Figure Source Data [file 44318_2025_420_MOESM12_ESM.zip › Source_data_EV/EV2/2C/EV2C.pdf]

EV2 B

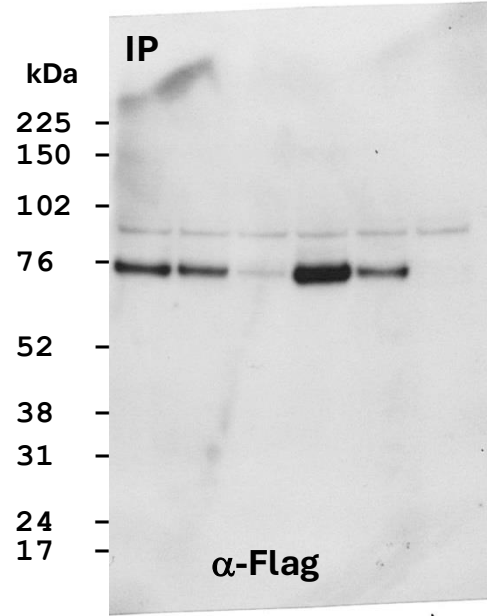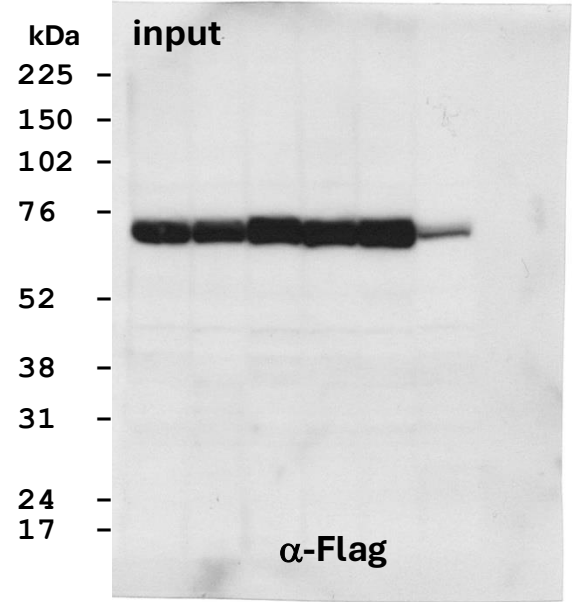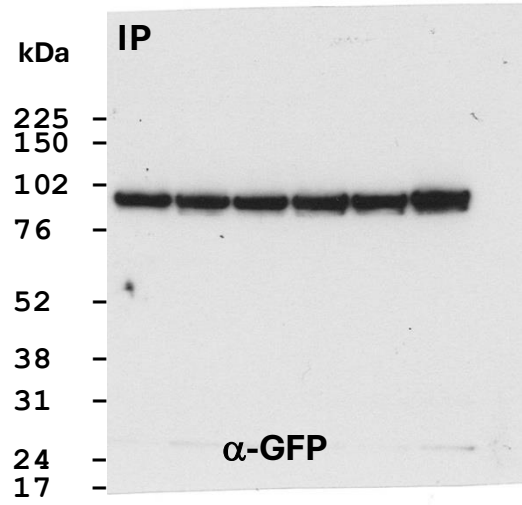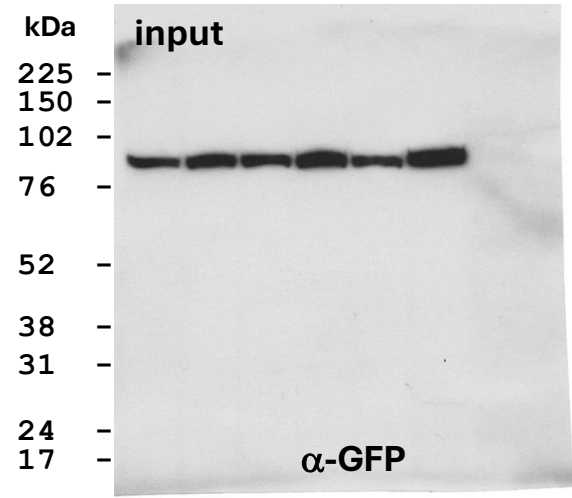

Supplement: Supplementary file 12 — EV Figure Source Data [file 44318_2025_420_MOESM12_ESM.zip › Source_data_EV/EV2/2B/EV2B.pdf]

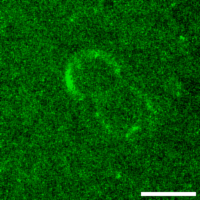

Supplement: Supplementary file 12 — EV Figure Source Data [file 44318_2025_420_MOESM12_ESM.zip › Source_data_EV/EV5/5I/ImageData Mud.tif]

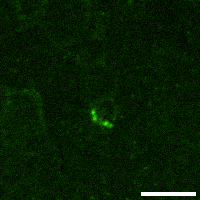

Supplement: Supplementary file 12 — EV Figure Source Data [file 44318_2025_420_MOESM12_ESM.zip › Source_data_EV/EV5/5A/ImageData Mud.tif]

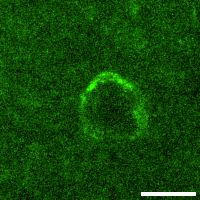

Supplement: Supplementary file 12 — EV Figure Source Data [file 44318_2025_420_MOESM12_ESM.zip › Source_data_EV/EV5/5F/ImageData Mud.tif]

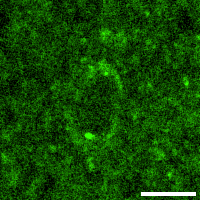

Supplement: Supplementary file 12 — EV Figure Source Data [file 44318_2025_420_MOESM12_ESM.zip › Source_data_EV/EV5/5H/ImageData Mud.tif]

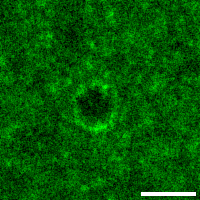

Supplement: Supplementary file 12 — EV Figure Source Data [file 44318_2025_420_MOESM12_ESM.zip › Source_data_EV/EV5/5E/ImageData Mud.tif]

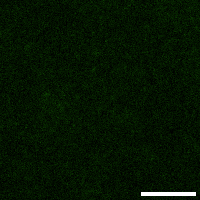

Supplement: Supplementary file 12 — EV Figure Source Data [file 44318_2025_420_MOESM12_ESM.zip › Source_data_EV/EV5/5B/ImageData Mud.tif]

EV4 C

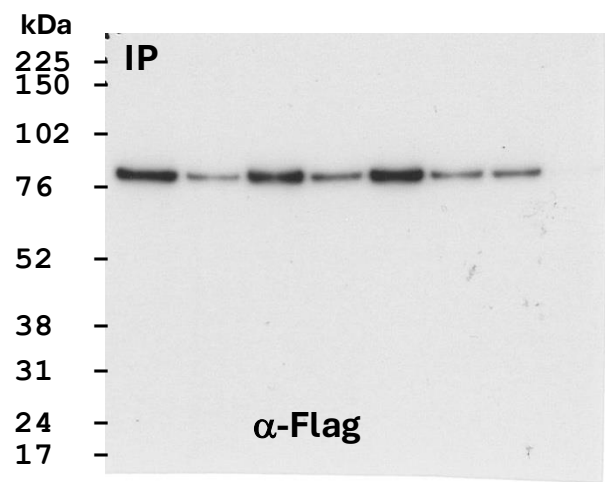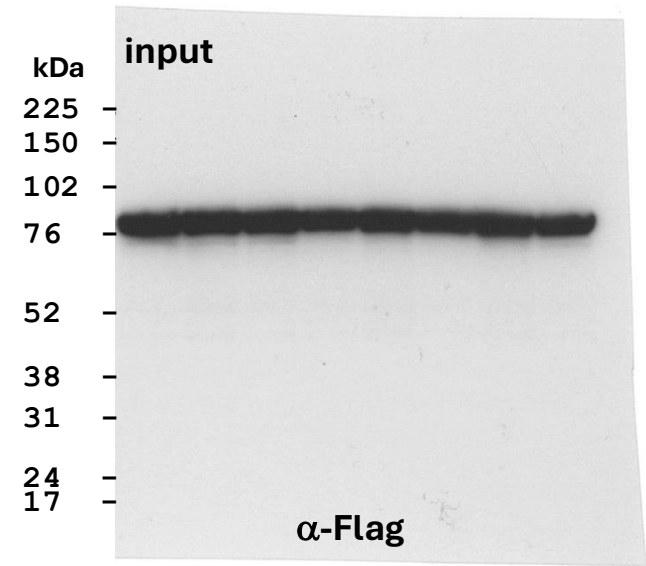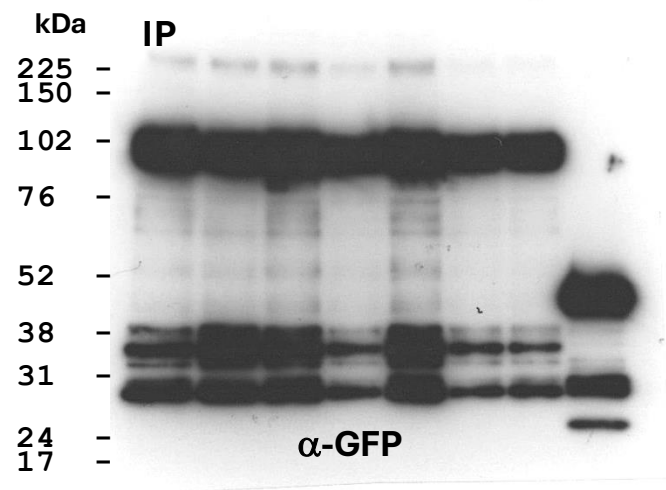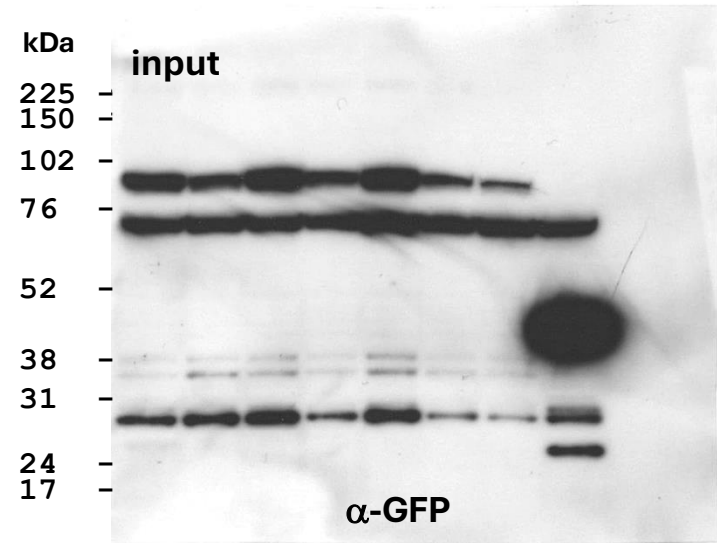

Supplement: Supplementary file 12 — EV Figure Source Data [file 44318_2025_420_MOESM12_ESM.zip › Source_data_EV/EV4/4C/EV4C.pdf]

EV4 A

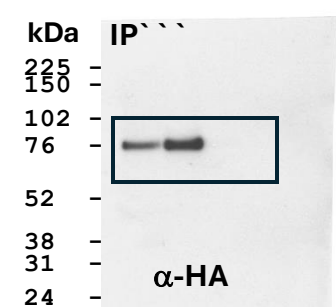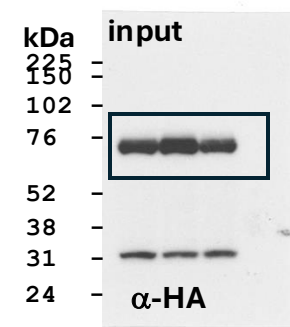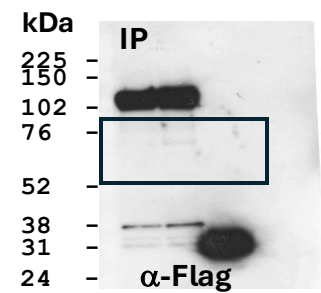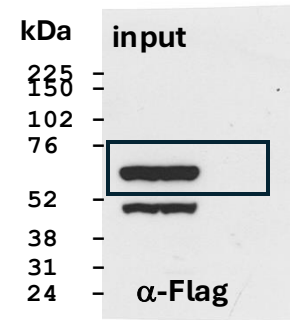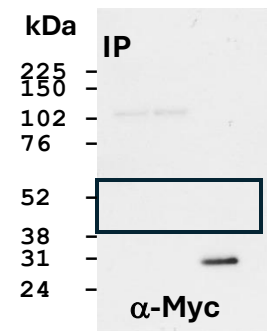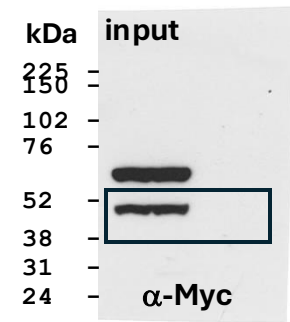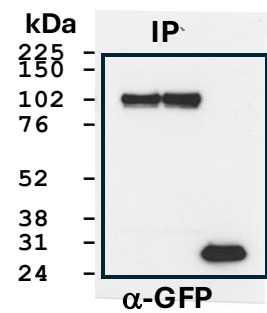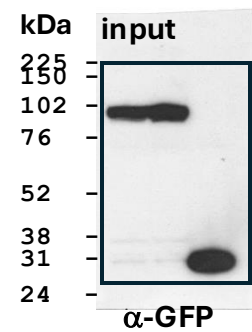

Supplement: Supplementary file 12 — EV Figure Source Data [file 44318_2025_420_MOESM12_ESM.zip › Source_data_EV/EV4/4A/EV4A.pdf]

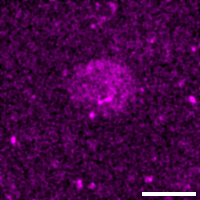

Supplement: Supplementary file 12 — EV Figure Source Data [file 44318_2025_420_MOESM12_ESM.zip › Source_data_EV/EV3/3B/ImageData Meru -18mins.tif]

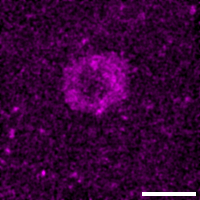

Supplement: Supplementary file 12 — EV Figure Source Data [file 44318_2025_420_MOESM12_ESM.zip › Source_data_EV/EV3/3C/ImageData Meru -18mins.tif]
