# Supplementary material for: Meru co-ordinates spindle orientation with cell polarity and cell cycle progression
Source: EMBO J. 2025 Apr 1;44(10):2949–75. doi: 10.1038/s44318-025-00420-5 (PMC12084343; doi:10.1038/s44318-025-00420-5)
Supplement: Supplementary file 13 — Expanded View Figures [file 44318_2025_420_MOESM13_ESM.pdf]

Expanded View Figures

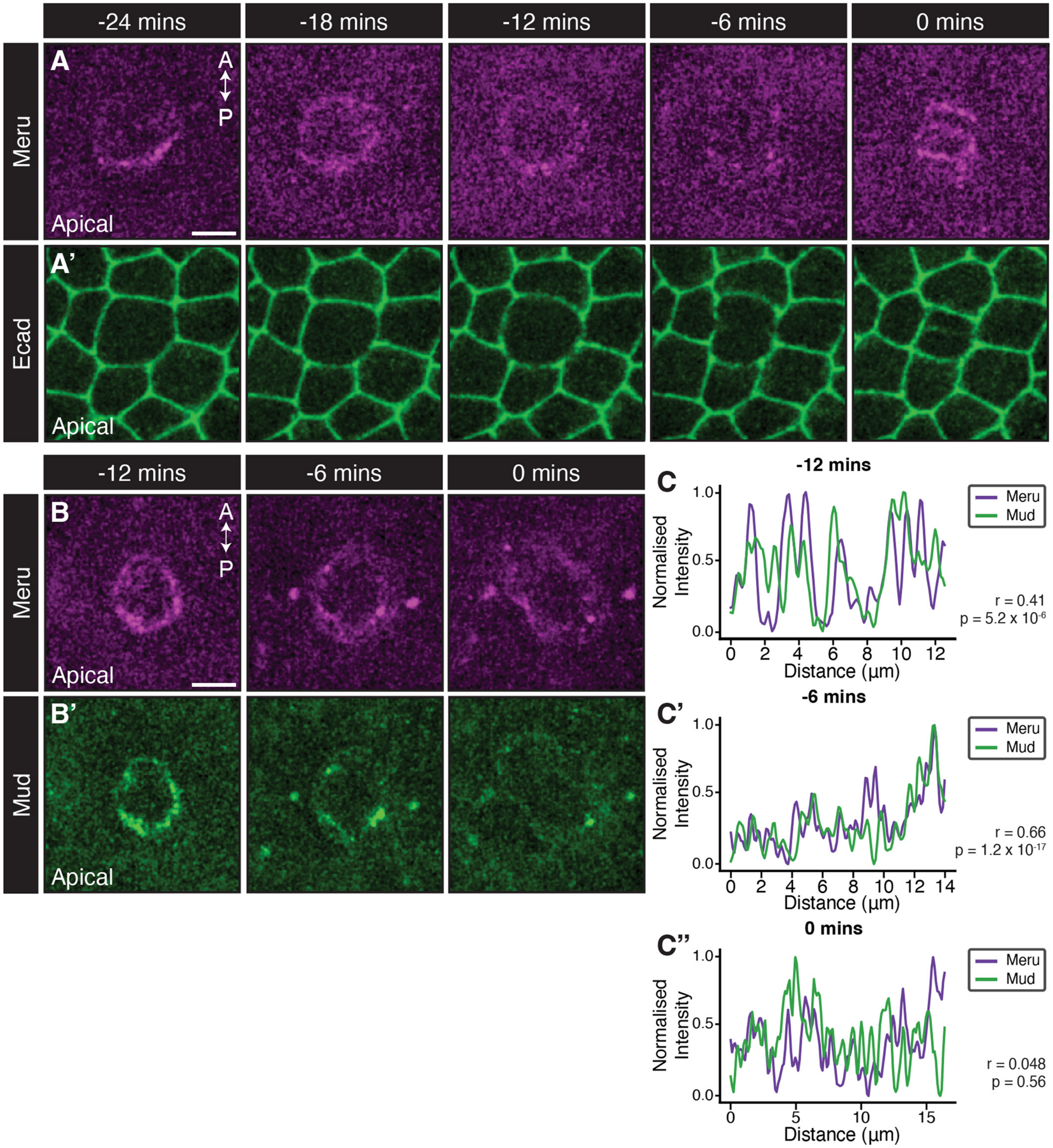

Figure EV1. Posterior-apical localisation of Meru occurs prior to SOP mitosis and persists throughout division.

(**A-B′**) Maximum intensity projections of apical-most three sections of pupal nota live-imaged on a confocal microscope at 16 h APF, 0 min marking the first frame indicating cytokinesis, of *neurG4 > UAS-mK2-meru* (magenta; **A**, **B**, see composite in Fig. 1D), co-expressed with Ecad-GFP and Mud-GFP (green; **A′**, **B′**, respectively). (**C–C″**) Single brightest slice in (**B**, **B′**) plotted as line graphs by measuring the grey value across the posterior domain as defined by Meru localisation normalised to highest value in each channel. High Pearson's coefficient (*r*) indicates positive correlation. *P* values calculated using a two-tailed test. Scale bars = 5 µm. Source data are available online for this figure.

 © The Author(s)

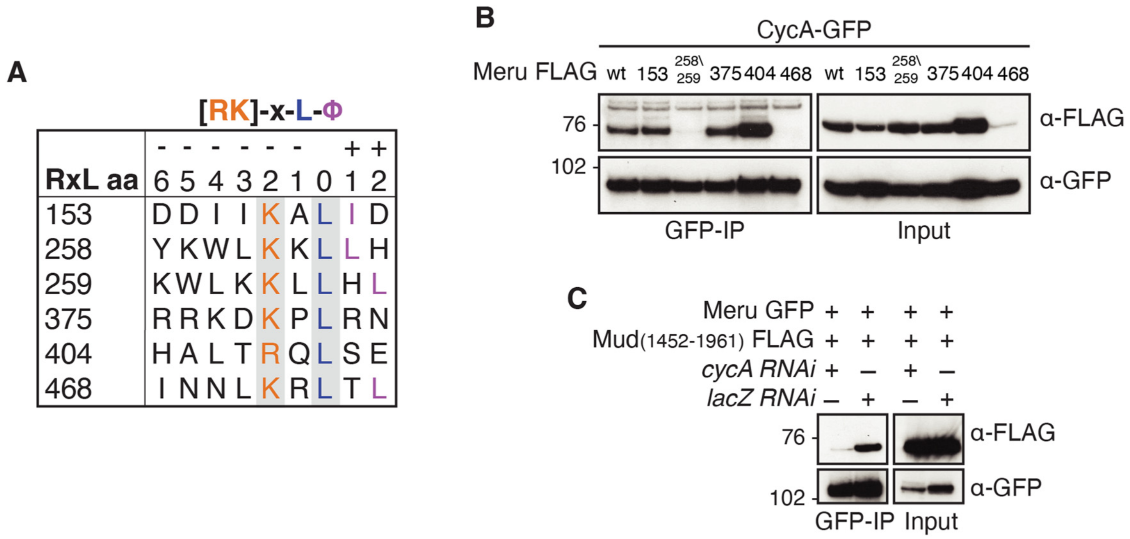

Figure EV2. The RxL motifs of Meru.

(**A**) The amino acid (aa) positions of the six RxL motifs identified in the Meru sequence are indicated on the left. On the right is the sequence alignment of all RxL motifs. Orange indicates the R/K and the L is labelled in blue at position 0. The hydrophobic aa is marked in purple and the grey boxes indicate the two aa that were mutated to Alanine in RxL mutants. (**B**, **C**) Western blots of co-IP experiment using cell lysates from transfected S2 cells, immunoprecipitated and probed using the indicated antibodies. (**B**) CycA immunoprecipitates with each Meru RxL motif mutant, except when Meru 258/9 is mutated to Alanines. (**C**) CycA depletion by RNAi reduced Meru/Mud association. Source data are available online for this figure.

© The Author(s)

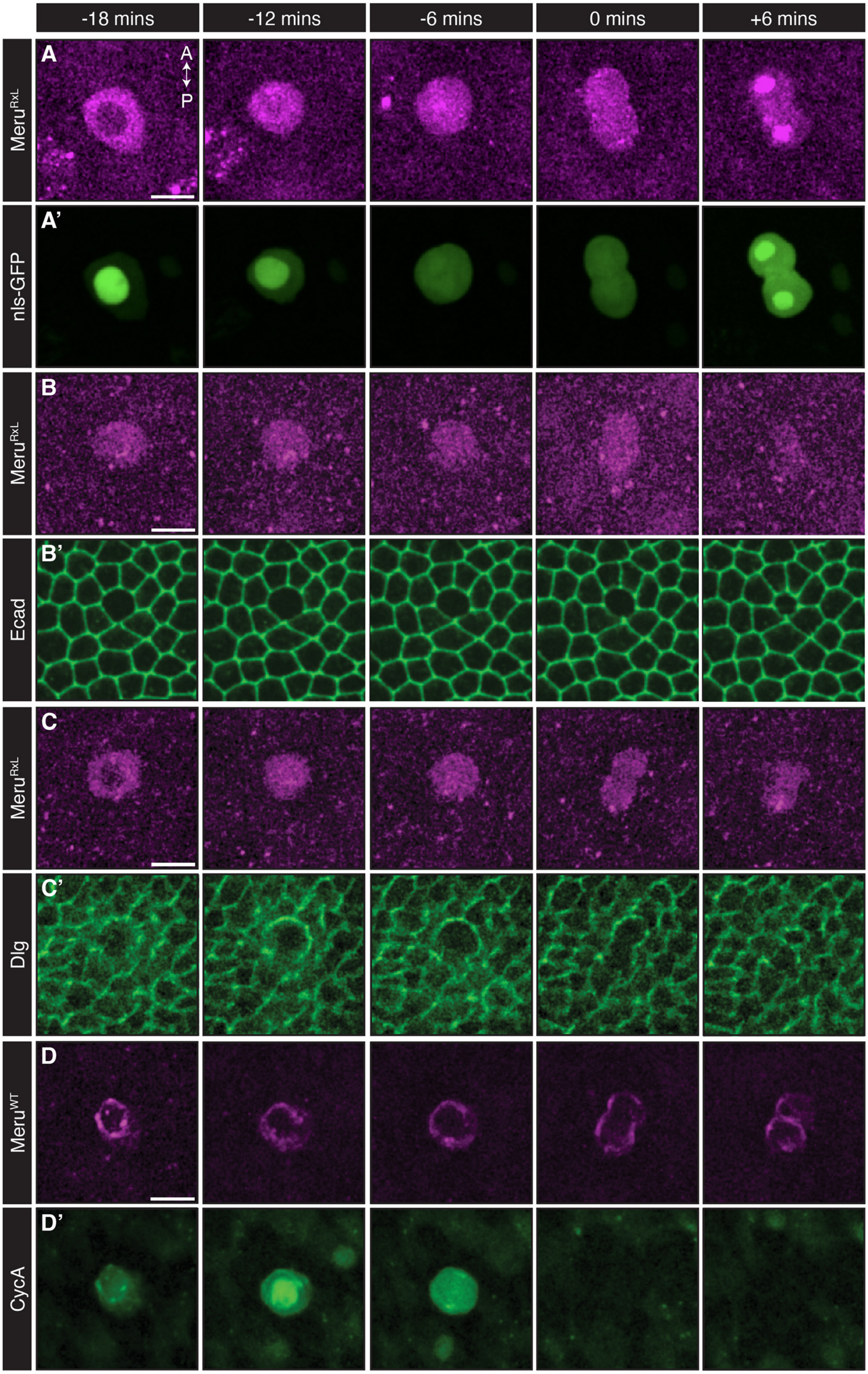
-18 mins
-12 mins
-6 mins
0 mins
+6 mins
A
A
P
Meru^RxL
A'
nls-GFP
B
Meru^RxL
B'
Ecad
C
Meru^RxL
C'
Dlg
D
Meru^WT
D'
CycA

 © The Author(s)

Figure EV3. 258/9 RxL motif mutant Meru no longer binds CycA and is mislocalised in vivo.

(**A–D**) Maximum intensity projections of pupal nota live-imaging at 16 h APF, 0 min marking the first frame indicating cytokinesis, of *neurG4 > UAS-mK2-meru*^{RxL} and *neurG4 > UAS-mK2-meru*^{WT} (magenta; **A–C** and **D**, respectively), co-expressed with nuclear nls-GFP, apical Ecad-GFP, basolateral Dlg-GFP and CycA-GFP (green; panels **A′**, **B′**, **C′** and **D′**, respectively). Scale bars = 10 µm. Source data are available online for this figure.

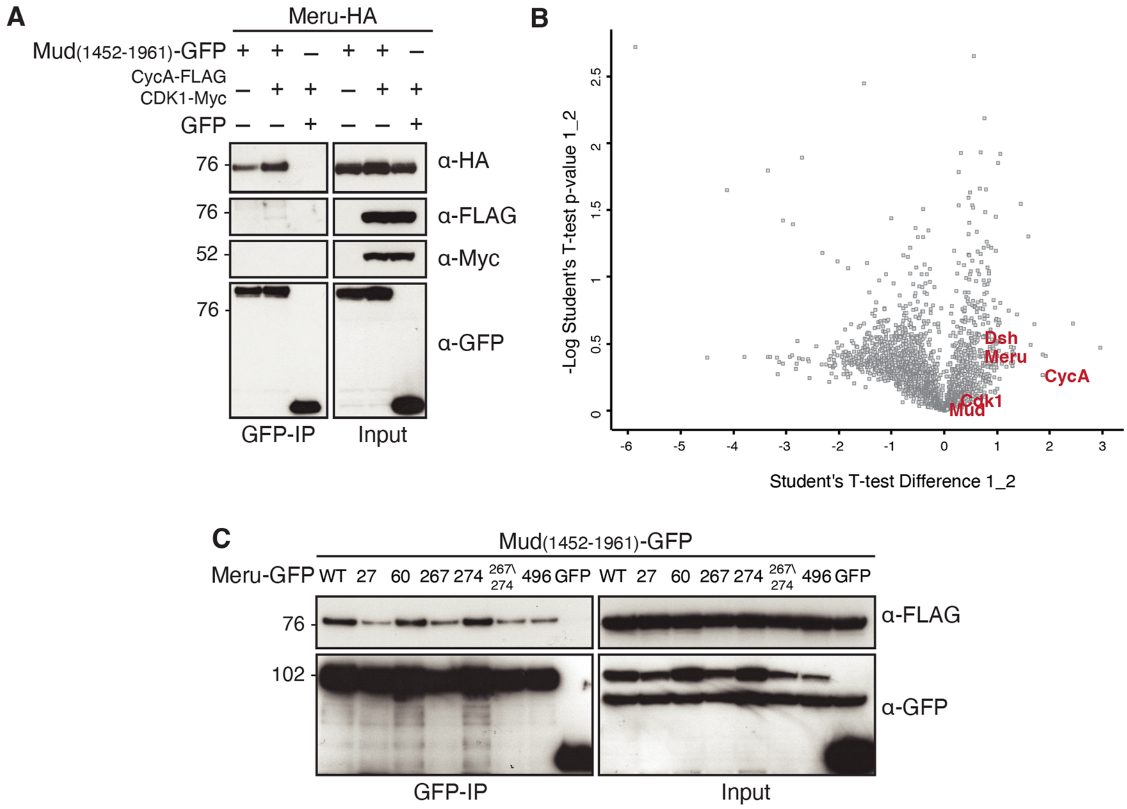

Figure EV4. Overexpression of Cdk1 and CycA promotes the Meru/Mud interaction.

(**A**, **C**) Western blots of co-IP experiment using cell lysates from transfected S2 cells, immunoprecipitated and probed using the indicated antibodies. (**A**) Overexpression of Cdk1/CycA increases the amount of Meru binding to Mud. (**B**) MeruRxL has decreased interaction with CycA, Cdk1, Mud and Dsh relative to MeruWT in a peptide identification analysis by MS (N = 2). (**C**) No single Meru S/T-P phosphosite is essential to co-IP with C-terminal Mud. Source data are available online for this figure.

 © The Author(s)

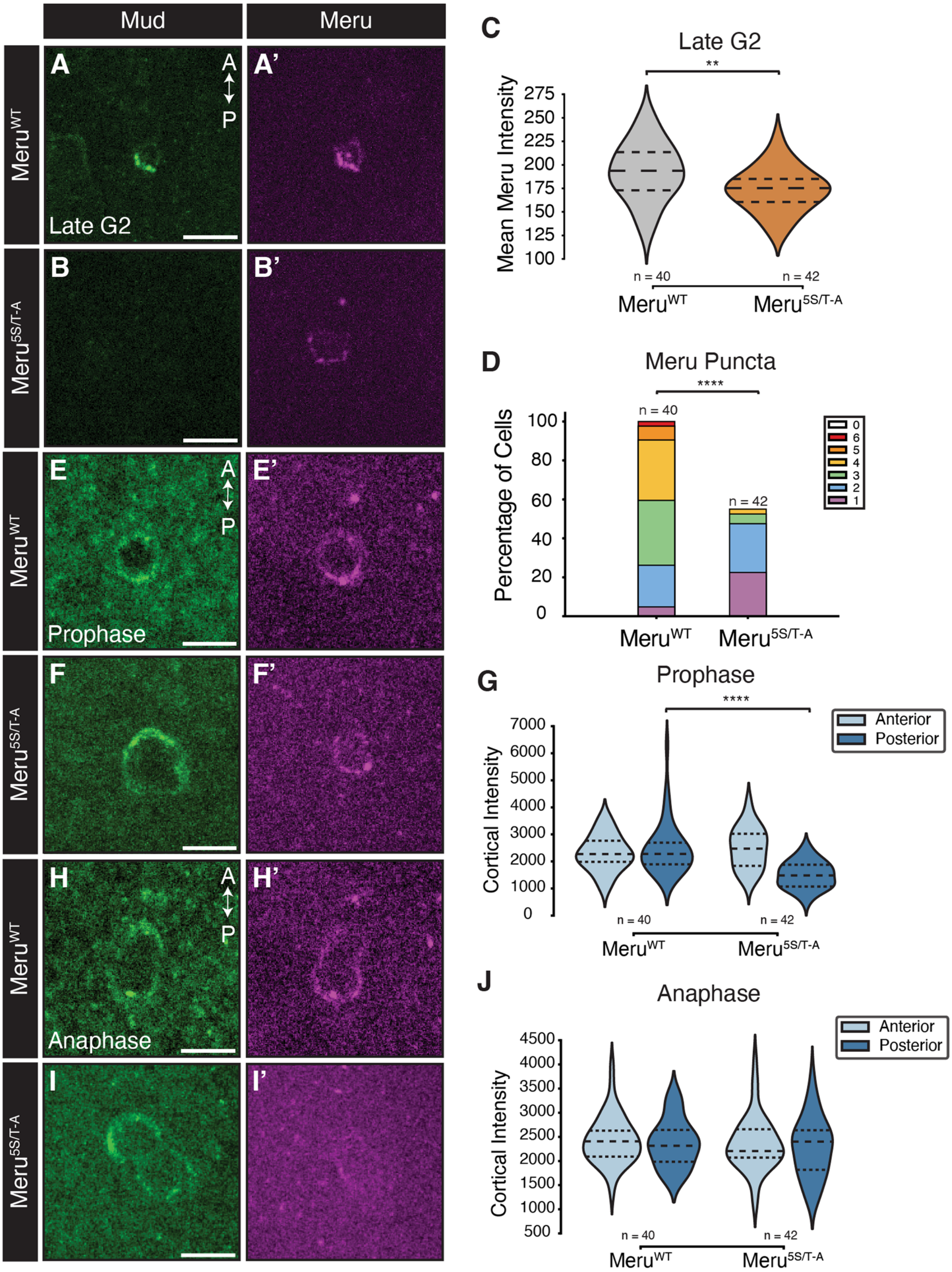
Mud
Meru
Meru^WT
Meru^5S/T-A
A
A'
A ↕ P
Late G2
B
B'
E
E'
Prophase
F
F'
H
H'
Anaphase
I
I'
C
Late G2
**
Mean Meru Intensity
n = 40
n = 42
Meru^WT
Meru^5S/T-A
D
Meru Puncta
****
Percentage of Cells
0
6
5
4
3
2
1
G
Prophase
****
Cortical Intensity
Anterior
Posterior
J
Anaphase

Figure EV5. Posterior cortical Mud levels return by anaphase in Meru$^{5S/T\text{-}A}$ mutants.

(**A–B′**) Single slice from confocal live-imaging of pupal nota at 16 h APF expressing *Mud-GFP* (green; **A**, **B**) and *neurG4 > UAS-mK2-meru^{WT}* (magenta; **A′**) or *neurG4 > UAS-mK2-meru$^{5S/T\text{-}A}$* (magenta; **B′**) at late G2. (**C**) Graph showing the mean intensity of posterior cortical Meru during late G2 in Meru^{WT} and Meru$^{5S/T\text{-}A}$ ($P = 2.7 \times 10^{-3}$). (**D**) Graph displaying the average number of Meru puncta at the posterior cortex in late G2. Puncta of the Meru$^{5S/T\text{-}A}$ mutant are significantly decreased relative to Meru^{WT} ($P = 9.10 \times 10^{-15}$). (**E–F′**) Maximum intensity projections (6 slices; **E**, **F**) from confocal live-imaging of pupal nota at 16 h APF expressing *Mud-GFP* (green; **E**, **F**) and *neurG4 > UAS-mK2-meru^{WT}* (magenta; **E′**) or *neurG4 > UAS-mK2-meru$^{5S/T\text{-}A}$* (magenta; **F′**) in a *meru^1* background at prophase. (**G**) Graph showing the intensity of cortical Mud at the anterior and posterior crescent of each genotype at prophase. Posterior Meru$^{5S/T\text{-}A}$ is significantly lower than Meru^{WT} ($P = 1.61 \times 10^{-7}$). (**H–I′**) Maximum intensity projections (6 slices; **H**, **I**) from confocal live-imaging of pupal nota at 16 h APF expressing *Mud-GFP* (green; **H**, **I**) and *neurG4 > UAS-mK2-meru^{WT}* (magenta; **H′**) or *neurG4 > UAS-mK2-meru$^{5S/T\text{-}A}$* (magenta; **I′**) in a *meru^1* background at anaphase. (**J**) Graphs showing the intensity of cortical Mud at the anterior and posterior crescent of each genotype at anaphase, where there is no measurable difference at anaphase ($P = 0.47$). $^{**}P < 0.01$ and $^{****}P < 0.0001$ using a Mann–Whitney *U* test. Large and small dashed lines in violin plots represent the median and Q1/Q3, respectively. Scale bars = 10 µm. (**C**, **D**, **G**, **J**) Number of SOPs imaged indicated on the panels from 3 nota. Source data are available online for this figure.

 © The Author(s)

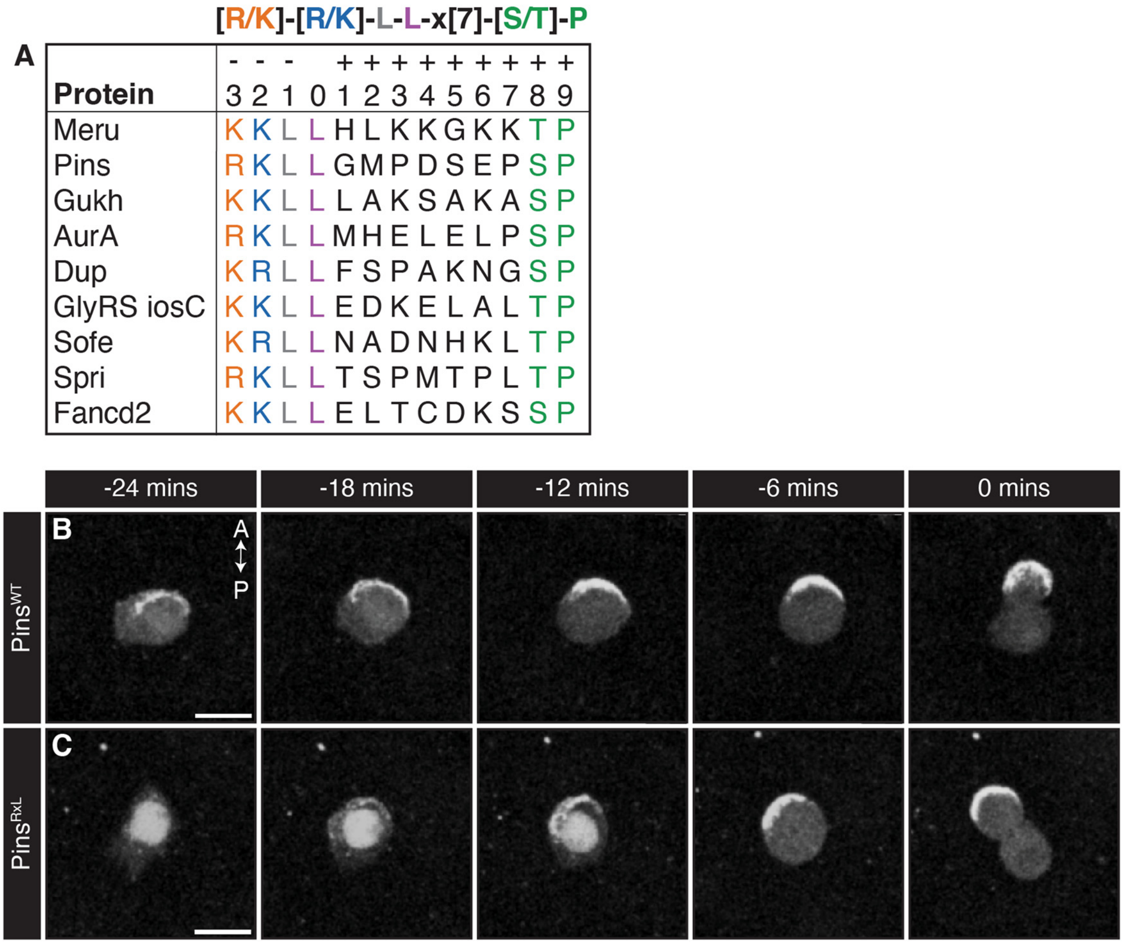

Figure EV6. Loss of the Pins RxL motif results in delayed cortical localisation.

(**A**) BLAST search results for novel [R/K]-[R/K]-L-L-x[7]-[S/T]-P (RRLL) motifs resulted in nine proteins of interest, including Meru, Pins, Gukh and AurA, all of which are involved in spindle orientation. (**B**, **C**) Maximum intensity projections from confocal live-imaging of pupal nota at 16 h APF, 0 min marking first frame in cytokinesis, shows expression of *UAS-mK2-Pins*^{WT} (**B**) and *UAS-mK2-Pins*^{RxL} (**C**) driven by *neurG4* during SOP mitosis. Scale bars = 10 µm. Source data are available online for this figure.
